# Spores of *Clostridium difficile* Clinical Isolates Display a Diverse Germination Response to Bile Salts

**DOI:** 10.1371/journal.pone.0032381

**Published:** 2012-02-22

**Authors:** Daniela Heeg, David A. Burns, Stephen T. Cartman, Nigel P. Minton

**Affiliations:** Clostridia Research Group, School of Molecular Medical Sciences, Centre for Biomolecular Sciences, University of Nottingham, Nottingham, United Kingdom; University of Connecticut, United States of America

## Abstract

*Clostridium difficile* spores play a pivotal role in the transmission of infectious diarrhoea, but in order to cause disease spores must complete germination and return to vegetative cell growth. While the mechanisms of spore germination are well understood in *Bacillus*, knowledge of *C. difficile* germination remains limited. Previous studies have shown that bile salts and amino acids play an important role in regulating the germination response of *C. difficile* spores. Taurocholate, in combination with glycine, can stimulate germination, whereas chenodeoxycholate has been shown to inhibit spore germination in a *C. difficile* clinical isolate. Our recent studies of *C. difficile* sporulation characteristics have since pointed to substantial diversity among different clinical isolates. Consequently, in this study we investigated how the germination characteristics of different *C. difficile* isolates vary in response to bile salts. By analysing 29 isolates, including 16 belonging to the BI/NAP1/027 type, we show that considerable diversity exists in both the rate and extent of *C. difficile* germination in response to rich medium containing both taurocholate and glycine. Strikingly, we also show that although a potent inhibitor of germination for some isolates, chenodeoxycholate does not inhibit the germination, or outgrowth, of all *C. difficile* strains. Finally, we provide evidence that components of rich media may induce the germination of *C. difficile* spores, even in the absence of taurocholate. Taken together, these data suggest that the mechanisms of *C. difficile* spore germination in response to bile salts are complex and require further study. Furthermore, we stress the importance of studying multiple isolates in the future when analysing the nutrients or chemicals that either stimulate or inhibit *C. difficile* spore germination.

## Introduction

*Clostridium difficile* is a Gram-positive, anaerobic spore former and the major underlying cause of hospital-acquired diarrhoea. *C. difficile* infection (CDI) is estimated to affect more than 500,000 people per year in the USA alone and the spread of CDI has led to patient isolation, ward closures and even hospital closures [1]. Infection with *C. difficile* may manifest as asymptomatic colonisation, but can also lead to severe diarrhoea that may then progress into a potentially fatal pseudo-membranous colitis [2]. Endospores, formed during sporulation, play a pivotal role in the transmission of disease. Spores shed in the faeces are able to withstand a variety of cleaning agents and can reside on hospital surfaces for prolonged periods of time [3]. Therefore, spores are regarded as the infectious stage of *C. difficile*. However, following ingestion by susceptible individuals, the progression to disease relies first on a return to vegetative cell growth through germination and then on production of the characteristic toxins [4].

Germination is defined as the irreversible loss of spore-specific characteristics and ultimately leads to vegetative cell growth. The mechanisms of germination have been studied extensively in *Bacillus spp.* in which nutrients and chemicals, termed germinants, can bind to specific receptors at the inner spore membrane [5]. At this point, the spore becomes committed to germination and subsequent events include release of monovalent cations (H^+^, K^+^ and Na^+^) and the spores' large depot of calcium dipicolinic acid (CaDPA) [6]. This redistribution of ions and water in the spore core likely precedes the activation of specific lytic enzymes that degrade the spore cortex, a thick layer of peptidoglycan differing subtly from vegetative cell peptidoglycan [7], [8]. Following cortex degradation, the spore becomes fully rehydrated, which in turn allows for a return to enzyme activity, metabolism and, finally, vegetative cell growth. To date, germination has been studied in several spore formers, including some *Clostridium spp.*, in which germinant receptors have been identified [9]–[11]. However, in *C. difficile* the absence of homologues to known germinant receptors has severely limited research into the mechanisms of germination in this species [12]–[14].

The mechanisms by which *C. difficile* spores sense a suitable environment for germination have not yet been investigated in great depth. However, recent work has revealed that bile salts play a pivotal role. It has been shown that *C. difficile* spores can germinate in response to the secondary bile salt taurocholate, which acts as a co-germinant with glycine [15], [16], and there is now evidence to suggest that, besides glycine, further amino acids may also act as co-germinants in combination with taurocholate [17], [18]. Furthermore, recent studies by Sorg and Sonenshein have described the role of the primary bile salt chenodeoxycholate in the inhibition of spore germination in a *C. difficile* clinical isolate [19], [20]. This adds an interesting element to the model of *C. difficile* colonisation in the gut. Increasing the concentration of chenodeoxycholate has been shown to reduce the efficacy of taurocholate as a germinant while, similarly, an increase in the concentration of taurocholate has been shown to reduce the inhibitory effect of chenodeoxycholate on germination [20]. However, spores of *C. difficile* are suggested to have a higher affinity for chenodeoxycholate than for taurocholate [20]. Therefore, in equal concentrations both cholate and chenodeoxycholate derivatives may compete for binding to putative *C. difficile* germinant receptors and germination may be inhibited. However, as the rate of absorption of chenodeoxycholate by the colon is ten times that of cholate [21], spores reaching the large intestine encounter a higher concentration of cholate derivatives, such as taurocholate. This suggests that germination may be inhibited until *C. difficile* spores reach the anaerobic environment of the large bowel, where conditions are suitable for vegetative cell growth.

The recent emergence of so-called ‘hypervirulent’ strains of *C. difficile*, such as those belonging to restriction endonuclease type BI, North American pulsed-field type 1, and PCR-ribotype 027 (BI/NAP1/027), presents a continuing challenge in healthcare settings worldwide. Strains belonging to this type are becoming increasingly common among clinical isolates, with some of these strains thought to be associated with more severe disease and an expanded repertoire of antibiotic resistance. As a result, understanding how these strains might differ from strains less frequently associated with ‘severe’ disease is of interest [22], [23]. Some strains of the BI/NAP1/027 type are believed to produce higher levels of toxin *in vitro* [24] and a number of studies have also concluded that this type is more prolific in terms of sporulation *in vitro* than other *C. difficile* types [25]–[30]. However, recent studies have now shown that BI/NAP1/027 strains do not have an increased sporulation rate compared with other types and these studies have also indicated that significant diversity exists in sporulation rates within the BI/NAP1/027 type [31], [32]. Interestingly, our previous studies of *C. difficile* sporulation rates have also indicated that the proportion of spores completing germination to form colonies may vary significantly among different isolates, suggesting that differences may exist in the germination response of different *C. difficile* strains to taurocholate [31], [32]. As germination is a prerequisite for colonisation, toxin production and subsequent disease, it is desirable to discover if germination characteristics vary among important clinical isolates. Furthermore, if variation exists in the germination response of different *C. difficile* isolates to taurocholate, it is conceivable that differences may also exist in the inhibitory effect of chenodeoxycholate on *C. difficile* spore germination.

Here, we sought to investigate the germination and outgrowth characteristics of different *C. difficile* isolates (Table 1) in rich medium containing chenodeoxycholate. We then examined the differences in the germination response to a medium containing both taurocholate and glycine. Finally, we aimed to identify generic germination differences between BI/NAP1/027 and non-BI/NAP1/027 strains while also studying how the *C. difficile* germination response varies within the BI/NAP1/027 type. We observed substantial diversity in germination characteristics among our group of clinical isolates and, perhaps most importantly, noted that chenodeoxycholate does not inhibit the germination of all *C. difficile* isolates.

**Table 1:** *C. difficile* strains used in this study.

Strain name	PCR-ribotype	Country	Source/Reference
**9001966**	002	The Netherlands	Ed Kuijper
**G,08,0000780**	002	The Netherlands	Ed Kuijper
**630Δ** ***erm***	012	Laboratory strain	[37]
**8085054**	014	The Netherlands	Ed Kuijper
**8079089**	017	The Netherlands	Ed Kuijper
**675,1**	017	Romania	Ed Kuijper
**5108111**	027	The Netherlands	Ed Kuijper
**CDC 38**	027	USA	[38]
**M13042**	027	Canada	[38]
**DH1432**	027	London, Barnet, UK	Val Hall
**DH1834**	027	East of England, Ipswich, UK	Val Hall
**DH1466**	027	East Midlands, Northampton, UK	Val Hall
**DH1858**	027	NE England,Sunderland, UK	Val Hall
**DH478**	027	SW England, Taunton, UK	Val Hall
**DH361**	027	London, Lewisham, UK	Val Hall
**DH1329**	027	West Midlands, UK	Val Hall
**R20352**	027	Canada	Val Hall
**R12087**	027	Historical EU strain (mid 80 s)	Val Hall
**R20291**	027	Stoke Mandeville, UK	Jon Brazier
**26131**	027	Finland	Ed Kuijper
**26173**	027	Finland	Ed Kuijper
**05-1223-046**	027	Belgium	Ed Kuijper
**2016**	078	Ireland	Ed Kuijper
**7004578**	078	The Netherlands	Ed Kuijper
**7009825**	078	The Netherlands	Ed Kuijper
**CD 2315**	078	Hungary	Ed Kuijper
**R9764**	081	Cardiff, UK	Jon Brazier/Ed Kuijper
**R12801**	106	Bristol, UK	Val Hall
**R10459**	106	Cardiff, UK	Jon Brazier/Ed Kuijper

## Results

### Chenodeoxycholate does not inhibit colony formation from spores of all *C. difficile* isolates

**Figure 1:**
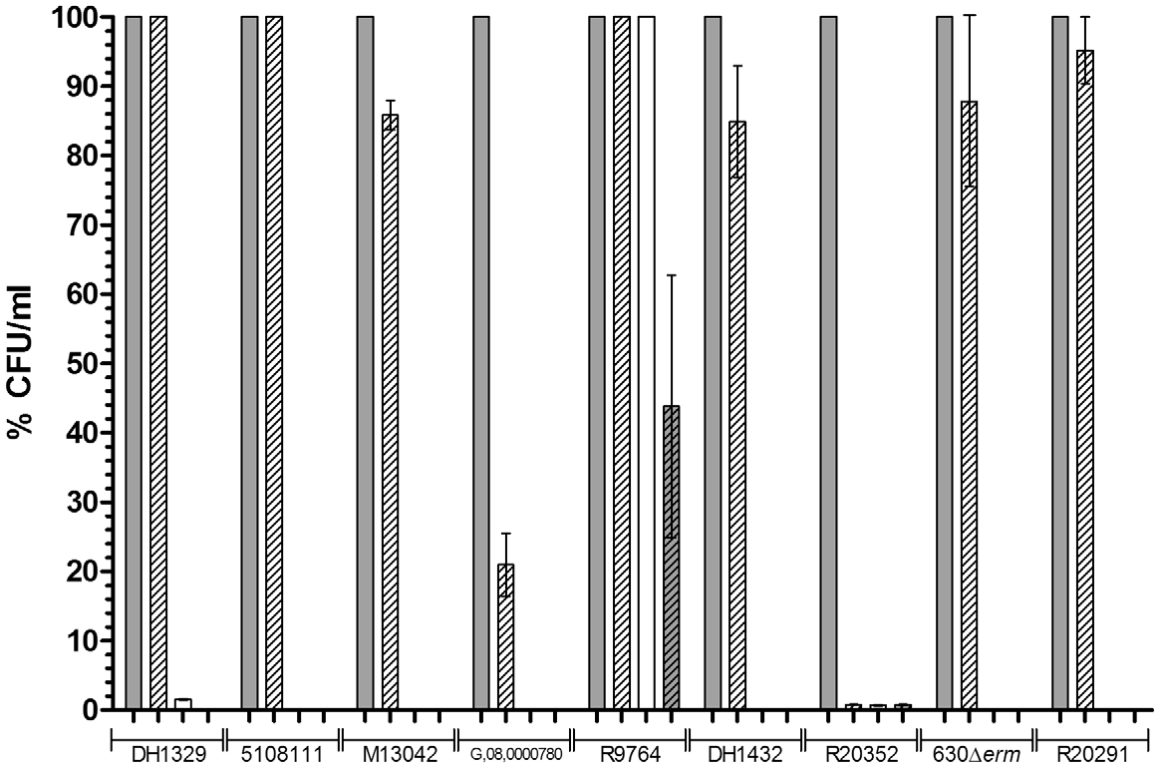
The CFU/ml formed on BHIS agar by nine *C. difficile* isolates after incubation with germinants. Spores of *C. difficile* were heat treated at 60°C for 25 min. CFU/ml were then enumerated on BHIS agar after 2 h incubation of spores with either i) BHIS supplemented with 0.1% (w/v) taurocholate (filled bars); ii) BHIS supplemented with 0.1% (w/v) taurocholate and 2 mM chenodeoxycholate (empty bars, diagonal lines); iii) BHIS alone (empty bars); or iv) dH_2_O (filled bars, diagonal lines). Colony formation was expressed as the percentage of CFU/ml observed compared to the CFU/ml observed after incubation with the positive control, BHIS supplemented with taurocholate. The data represent the average of three independent experiments and error bars indicate the standard errors of the means.

Chenodeoxycholate has previously been shown to inhibit spore germination in a *C. difficile* clinical isolate [19], [20]. As our previous work has indicated that differences may exist in the germination properties of different *C. difficile* isolates [31], [32], we elected to analyse if chenodeoxycholate inhibits the spore germination of nine isolates. Our initial investigations focused on the ability of dormant spores to return to vegetative cell growth following incubation with chenodeoxycholate. Accordingly, we measured the number of colonies recovered on BHIS agar following incubation of spores in BHIS medium supplemented with either 0.1% (w/v) taurocholate alone, or 0.1% (w/v) taurocholate further supplemented with 2 mM chenodeoxycholate (a concentration previously shown to inhibit germination of *C. difficile* spores and similar to the concentration of bile that enters the large intestine) [19], [33]. As negative controls, colony-forming units (CFU) were also enumerated on BHIS agar after incubation of spores in BHIS and dH_2_O, separately. We observed that chenodeoxycholate either completely or partially inhibited colony formation (i.e. the result of germination and outgrowth) from spores in six of the tested isolates (Fig. 1). When incubated with either i) BHIS supplemented with taurocholate and chenodeoxycholate; ii) BHIS alone; or iii) dH_2_O, R20352 was found to produce 100-fold fewer CFU/ml compared with taurocholate incubation. Five of the tested isolates (G,08,0000780, M13042, DH1432, 630Δ*erm*, and R20291) were able to form colonies following incubation with chenodeoxycholate, although not necessarily to the same level as the CFU/ml observed after incubation with taurocholate alone (21%, 85%, 85%, 88%, and 95%, respectively compared with taurocholate alone). Additionally, three of the tested isolates (DH1329, 5108111, and R9764) were able to produce CFU comparable to the number observed following incubation with taurocholate alone (i.e. the positive control) (Fig. 1). Interestingly, we observed that R9764 (PCR-ribotype 081) was able to produce CFU when incubated with both BHIS alone and dH_2_O alone (100% and 44%, respectively compared to taurocholate only). The observation of colony formation following incubation in dH_2_O alone can likely be explained by the fact that the spore suspensions were subsequently spread onto BHIS agar, where germination could then have occurred. These data suggest that chenodeoxycholate does not inhibit spore germination in all *C. difficile* strains. Furthermore, they also suggest that spores of some isolates of *C. difficile* are able to germinate in the absence of bile salts, in BHIS alone.

### Chenodeoxycholate does not inhibit the germination of all *C. difficile* isolates

**Figure 2:**
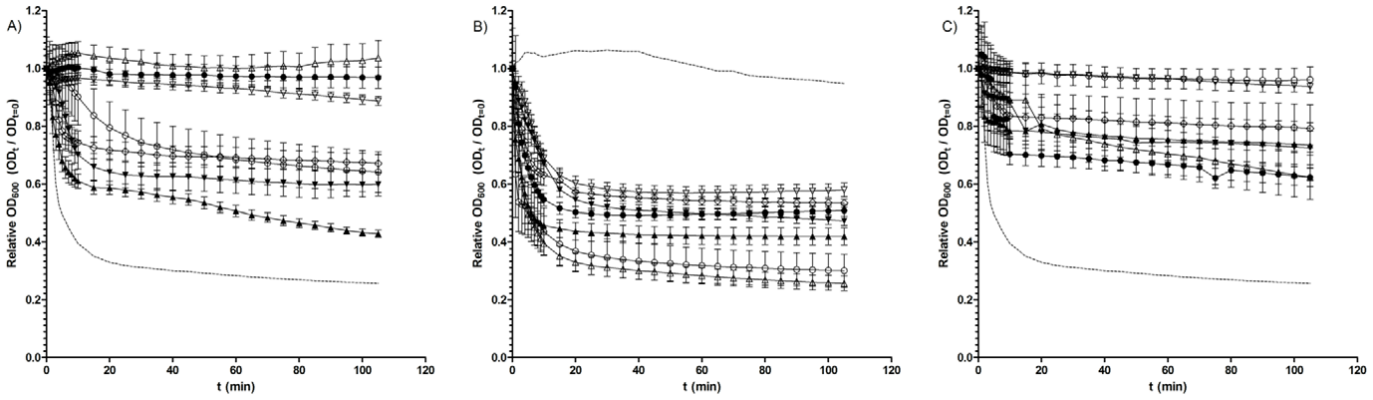
Loss of spore OD_600_ of *C. difficile* isolates during incubation in A) BHIS supplemented with taurocholate and chenodeoxycholate; B) BHIS supplemented with taurocholate; or C) BHIS alone. *C. difficile* spores were heat treated at 60°C for 25 min and then incubated in BHIS with or without supplement. Germination was expressed as the loss of spore OD_600_. A) Loss of OD_600_ of spores of *C. difficile* isolates incubated in BHIS supplemented with 0.1% (w/v) taurocholate and 2 mM chenodeoxycholate. ▴, DH1834; ▾, 5108111; •, 9001966; ▵, 26131; ○, 7004578; ◊, CD 2315; and ▿, CDC38. The dotted line represents the loss of OD_600_ of the positive control strain (05-1223-046) in response to BHIS supplemented with 0.1% (w/v) taurocholate alone. B) Loss of OD_600_ of spores of *C. difficile* isolates incubated with BHIS supplemented with 0.1% (w/v) taurocholate. ▴, DH1834; ▾, 5108111; •, DH1432; ▵ 05-1223-046; □, CD 2315; ▿, CDC 38; and ○, DH1858. The dotted line represents the negative control strain (DH361) during incubation with both 0.1% (w/v) taurocholate and 2 mM chenodeoxycholate (a strain found, in preliminary studies, not to germinate when exposed to chenodeoxycholate). C) Loss of OD_600_ of spores of *C. difficile* isolates incubated with BHIS alone. ▴, DH1834; ▾, 5108111; •, 8085054; ▵, 05-1223-046; ○, 26173; and ▿, CDC38. The dotted line represents the loss of OD_600_ of the positive control strain (05-1223-046) in response to BHIS supplemented with 0.1% (w/v) taurocholate alone. All data represent the average of three independent experiments and error bars indicate the standard errors of the means.

**Figure 3:**
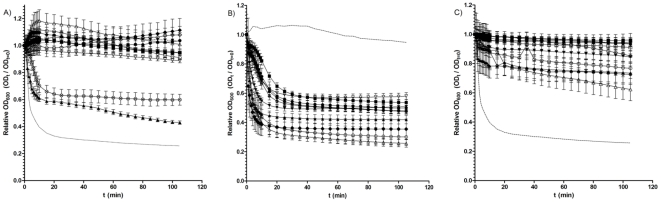
Loss of spore OD_600_ of 11 *C. difficile* BI/NAP1/027 isolates during incubation in A) BHIS supplemented with taurocholate and chenodeoxycholate; B) BHIS supplemented with taurocholate; or C) BHIS alone. A), B), and C) ▴, DH1834; ▾, DH1432; ▪, 26173; •, 26131; ♦, DH478; ▵, 05-1223-046; □, DH361; ○, DH1858; ◊, DH1466; ▿, CDC38; and <$>\raster="rg4"<$>, 5108111. A) The dotted line represents the loss of OD_600_ of the positive control strain (05-1223-046) in response to BHIS supplemented with 0.1% (w/v) taurocholate alone. B) The dotted line represents the negative control strain (DH361) during incubation with both 0.1% (w/v) taurocholate and 2 mM chenodeoxycholate (a strain found, in preliminary studies, not to germinate when exposed to chenodeoxycholate). C) The dotted line represents the loss of OD_600_ of the positive control strain (05-1223-046) in response to BHIS supplemented with 0.1% (w/v) taurocholate alone. All data represent the average of three independent experiments and error bars indicate the standard errors of the means.

Following the observation that spores of some *C. difficile* isolates can complete germination and form colonies in the presence of chenodeoxycholate, we reasoned that the germination response of *C. difficile* spores to chenodeoxycholate may vary. Consequently, we analysed how the initiation of germination varied when spores of 15 different *C. difficile* isolates were exposed to chenodeoxycholate. We expressed germination as the loss of optical density at 600 nm (OD_600_), a known indicator of bacterial spore germination [20], [34]. Accordingly, we measured the loss of OD_600_ in response to BHIS supplemented with both 0.1% (w/v) taurocholate and 2 mM chenodeoxycholate. Interestingly, we observed that while the germination of most isolates appeared to be inhibited by chenodeoxycholate, this was not the case with all of the isolates tested (Fig. 2A and Table 2). Although no isolate germinated to the same degree as the germination positive control (05-1223-046, a strain found, in preliminary studies, to exhibit a 74% drop in OD_600_ in response to taurocholate), a number of isolates, DH1834 (57%), 5108111 (40%), 7004578 (36%) and CD 2315 (33%) were able to germinate in the presence of chenodeoxycholate. Interestingly, when analysing 11 BI/NAP1/027 isolates, two (DH1834, and 5108111) were able to germinate even in the presence of chenodeoxycholate, while germination appeared to be inhibited in spores of the other nine isolates (Fig. 3A). These data suggest that, despite being a potent inhibitor of spore germination in many *C. difficile* isolates (over two-thirds of our entire sample set) chenodeoxycholate does not universally inhibit spore germination in all *C. difficile* strains. Furthermore, variation appears to exist in the extent of chenodeoxycholate inhibition of germination within the BI/NAP1/027 type.

**Table 2:** Percentage loss of spore optical density after incubation of *C. difficile* spores with bile salts for 2 h.

Strain name	PCR-ribotype	0.1% taurocholate	2 mM chenodeoxycholate	BHIS
**9001966**	002	47% (0.529)	3% (0.968)	11% (0.894)
**8085054**	014	72% (0.376)	13% (0.868)	38% (0.623)
**5108111**	027	53% (0.473)	40% (0.599)	27% (0.725)
**CDC 38**	027	42% (0.580)	12% (0.888)	6% (0.938)
**DH1432**	027	49% (0.508)	0% (1.031)	15% (0.846)
**DH1834**	027	58% (0.420)	57% (0.429)	27% (0.734)
**DH1466**	027	64% (0.355)	0% (1.079)	15% (0.852)
**DH1858**	027	70% (0.301)	9% (0.910)	23% (0.768)
**DH478**	027	52% (0.479)	0% (1.113)	5% (0.947)
**DH361**	027	58% (0.422)	5% (0.947)	9% (0.911)
**26131**	027	50% (0.500)	0% (1.036)	7% (0.934)
**26173**	027	46% (0.537)	5% (0.946)	4% (0.961)
**05-1223-046**	027	74% (0.257)	0% (1.009)	38% (0.623)
**7004578**	078	46% (0.536)	36% (0.644)	7% (0.926)
**CD 2315**	078	47% (0.534)	33% (0.671)	21% (0.792)

*C. difficile* spores were heat treated at 60°C for 25 min and then incubated with either i) BHIS supplemented with 0.1% (w/v) taurocholate; ii) BHIS supplemented with 0.1% (w/v) taurocholate and 2 mM chenodeoxycholate; or iii) BHIS alone. The data represent the average of three independent experiment and numbers in parentheses represent the mean of the final OD_600_ value.

### Diversity in the germination of *C. difficile* spores in response to taurocholate

We have previously reasoned that the proportion of spores that form colonies may vary from strain to strain, possibly due to differences in heat resistance and/or germination characteristics among different *C. difficile* isolates [31], [32]. When measuring the loss in OD_600_ of spores of 15 *C. difficile* isolates (of varying PCR-ribotypes) during incubation with BHIS supplemented with 0.1% (w/v) taurocholate (Fig. 2B and Table 2), we observed significant variation in the germination response among different *C. difficile* isolates (p<0.00001). Even though a maximal loss of OD_600_ was observed within the first 20 min for most isolates, the final percentage drop in spore OD_600_ was considerably different among our sample of isolates. Starting from a baseline adjusted OD_600_ of 1, the largest drop in OD_600_ was found to be 74% (05-1223-046), while the smallest drop was 42% (CDC 38). This indicates that different isolates of *C. difficile* may vary in both their rate and extent of germination in response to taurocholate.

**Figure 4:**
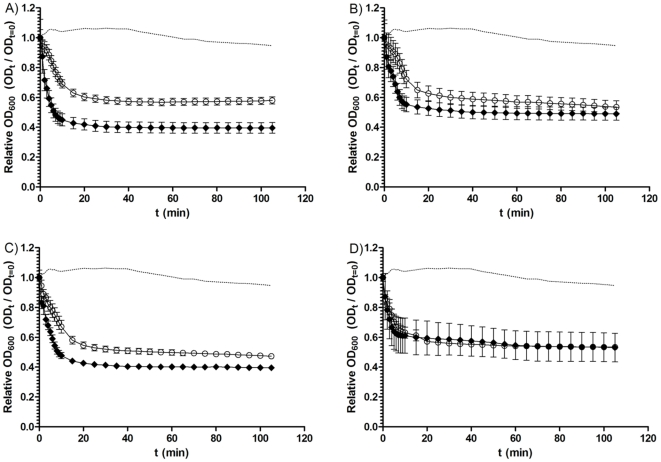
Loss of spore OD_600_ of four *C. difficile* isolates during incubation with different concentrations of taurocholate. *C. difficile* spores were heat treated at 60°C for 25 min and then incubated in BHIS supplemented with either 0.1% (w/v) taurocholate (○) or 1% (w/v) taurocholate (•) for 2 h. A) CDC38; B) 7004578; C) 5108111; D) CD2315. The data represent the average of three independent experiments and error bars indicate the standard errors of the means. The dotted line represents the negative control strain (DH361) during incubation with both 0.1% (w/v) taurocholate and 2 mM chenodeoxycholate (a strain found, in preliminary studies, not to germinate when exposed to chenodeoxycholate).

To evaluate if different concentrations of taurocholate alter the germination response of *C. difficile* spores, we incubated spores of four isolates with BHIS supplemented with either 0.1% or 1% (w/v) taurocholate (Fig. 4). Interestingly, we again observed differences among these isolates. For example, CD 2315 was found to germinate similarly in response to both concentrations of taurocholate. Conversely, CDC 38 appeared to germinate at a faster rate when incubated with 1% (w/v) taurocholate than when incubated with 0.1% (w/v) taurocholate and CDC 38 also appeared to show a larger drop in OD_600_ when exposed to 1% (w/v) taurocholate. This suggests that different isolates of *C. difficile* may vary in their response to different concentrations of germinants, which could be of interest for future studies.

In keeping with our previous work [31], [32], we also sought to investigate the germination characteristics of 16 BI/NAP1/027 isolates, independent of other PCR-ribotypes. Interestingly, when examining the germination properties of 11 of these BI/NAP1/027 isolates spectrophotometrically, we observed significant variation in both the rate and extent of germination within the BI/NAP1/027 group in response to taurocholate (p<0.0001), with the drop in OD_600_ ranging from 74% in strain 05-1223-046 to 42% in strain CDC 38 (Fig. 3B). These data support our hypothesis that strains grouped according to a particular typing method do not necessarily exhibit similar *in vitro* characteristics.

### Rich medium stimulates germination and outgrowth of *C. difficile* spores

**Figure 5:**
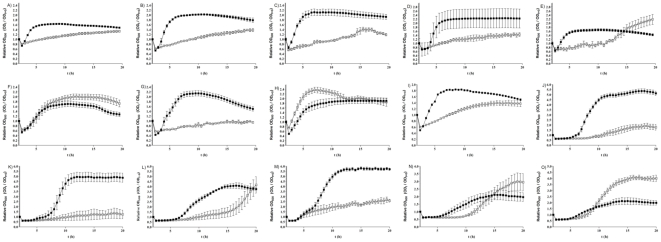
Cell growth from spores of 15 *C. difficile* isolates. Spores were heat treated at 60°C for 25 min and then incubated with either BHIS alone (□) or BHIS supplemented with 0.1% (w/v) taurocholate (▪). The change in OD_600_ was measured over 24 h. A) 9001966; B) R12087; C) R12801; D) 675,1; E) G,08,0000780; F) 7004578; G) 5108111; H) R9764; I) DH1329; J) 8079089; K) R10459; L) 7045389; M) R20352; N) 7009825; O) 2016. The data represent the average of three independent experiments and error bars indicate the standard errors of the means.

We observed above that spores of some *C. difficile* strains were able to form colonies on BHIS agar, even in the absence of known germinants such as taurocholate. To assess whether components of BHIS can act as germinants, spores of 15 *C. difficile* isolates were incubated with BHIS alone and the loss of OD_600_ was measured. Six isolates (8085054, 05-1223-046, DH1834, DH1858, 5108111 and CD 2315) of *C. difficile* showed a slow but significant (p<0.001) loss of OD_600_ (Fig. 2C and Table 2), suggesting that these isolates are able to germinate in response to BHIS alone. To investigate whether this germination would lead to vegetative cell growth, we next measured the change in OD_600_ of *C. difficile* cultures over a 20 h time period, during incubation of spores with either BHIS only or BHIS supplemented with 0.1% (w/v) taurocholate (Fig. 5). Once more, we observed considerable differences in the growth properties among strains. The isolates R12087, R12801, R10459, R20352, 9001966, 5108111, 8079089 and 675,1 were not able to grow during incubation with BHIS alone, while the isolates 7009825, 7045389, DH1329, 2016, 7004578, R9764 and G,08,0000780 were able to demonstrate cell growth without taurocholate supplement (Fig. 5). This suggests that components of rich media such as BHIS can induce germination of *C. difficile* spores, which can subsequently lead to vegetative cell growth.

## Discussion

Spores of *C. difficile* are pivotal for transmission of infection, but in order to cause disease spores must return to vegetative cell growth through germination. Bile salts likely play an important role in *C. difficile* germination *in vivo* and it has been shown previously that chenodeoxycholate can act as an inhibitor of germination *in vitro* [16], [19], [35]. Here, we examined how the germination response to bile salts varies among spores of 29 different *C. difficile* isolates. We found that, despite being a potent inhibitor of spore germination in many isolates, chenodeoxycholate did not inhibit the initiation of germination, or the subsequent vegetative cell outgrowth, in all isolates. We also observed substantial variation in both the rate and extent of germination in response to taurocholate among different *C. difficile* isolates, in addition to a possible concentration-dependent response to taurocholate. Furthermore, we provide evidence that components of a rich medium such as BHIS may be sufficient to induce germination of *C. difficile* spores, even in the absence of taurocholate.

Due to the proposed inhibitory effect on *C. difficile* spore germination, chenodeoxycholate has been suggested as a potential prophylactic drug for the prevention of *C. difficile* colonisation [20]. However, we have shown in this study that chenodeoxycholate does not appear to inhibit the spore germination of all *C. difficile* strains. Therefore, a drug based on chenodeoxycholate may not be sufficient for the control of CDI, as its inhibitory action on spore germination is unlikely to be specific to all strains of *C. difficile*. Given our evidence, we suggest that more studies regarding the germination mechanisms of *C. difficile* are needed in order to explore the true potential of germination-based therapeutic drugs. Although it has been suggested that there is kinetic evidence for the presence of germinant receptors [16], it is still unknown if germinants such as taurocholate bind to a receptor or simply diffuse through the spore and trigger the germination cascade. Consequently, it is difficult for us to speculate on what the molecular basis may be for the variation in germination characteristics that we have observed in this study.

We included 16 BI/NAP1/027 isolates in our study and observed significant diversity in the germination response to taurocholate among this group. We have previously found substantial variation in sporulation characteristics among *C. difficile* BI/NAP1/027 isolates [31], [32], [36]. As a result, the current study supports our hypothesis that isolates grouped according to a particular molecular typing technique cannot be assumed to have identical characteristics. To the contrary, this study indicates that general conclusions should not be made when discussing the BI/NAP1/027 type. The use of the term ‘hypervirulent’ should, therefore, perhaps be restricted to the description of individual isolates and should only be used when clearly defined and supported by appropriate clinical data.

In this study we also observed differences in the ease of preparing pure spore suspensions from different *C. difficile* isolates. Repeatedly washing spore suspensions in dH_2_O was sufficient for the preparation of pure spores from many *C. difficile* isolates. However, we noted that for some isolates it was not possible to obtain spore suspensions sufficiently free of vegetative cells and cell debris using this method. Furthermore, even when pure spore suspensions were prepared, excessive spore clumping in some isolates prevented the analysis of germination by the loss of spore OD_600_, as spore clumping results in a rapid loss of OD_600_ that may be misinterpreted as the onset of germination. For that reason, six of the isolates analysed for colony formation (M13042, G.08,0000780, R20352, R9764, 630Δ*erm*, R20291) were unable to be studied for loss of OD_600_. This is an interesting finding and perhaps reinforces our hypothesis that different *C. difficile* isolates exhibit varying *in vitro* characteristics. Studying these differences in more detail is a clear area for future studies.

We have uncovered differences in the *in vitro* germination response of different *C. difficile* clinical isolates, but it is still unclear how these different germination properties relate to *in vivo* germination characteristics. Given the likely importance of germination for *C. difficile* colonisation, it is interesting that different isolates appear to germinate at different rates in response to bile salts. One could speculate that, ideally, a balance of spores and vegetative cells may need to be maintained in order for *C. difficile* to effectively colonise the gut. Therefore, a specific germination frequency may not only result in a sufficient number of vegetative cells required for colonisation, but also in a number of spores that could resist therapeutically administered antibiotics, such that they could then cause a relapse in disease at a later stage. However, further investigation is warranted in order to test this hypothesis.

## Materials and Methods

### *C. difficile* isolates and growth conditions

A total of 29 isolates were chosen for analysis (Table 1), including 16 BI/NAP1/027 isolates and 13 non-BI/NAP1/027 isolates. The non-BI/NAP1/027 group included isolates of PCR-ribotypes 002, 003, 012, 014, 017 (n = 2), 078 (n = 4), 081, and 106 (n = 2). The chosen isolates included isolates from the UK (n = 11), the USA, Canada (n = 2), Hungary, Finland (n = 2), Belgium, Ireland, Romania, and the Netherlands (n = 7), in addition to an historical EU isolate. *C. difficile* 630Δ*erm* was derived previously through continuous subculturing of the 630 wild-type strain [37]. Unless otherwise stated, all *C. difficile* isolates were cultivated at 37°C, in an anaerobic workstation (Don Whitley, United Kingdom), in BHIS (brain heart infusion supplemented with yeast extract [5 mg/ml, Oxoid] and L-cysteine [0.1% (w/v), Sigma; United Kingdom]) broth or agar.

### Preparation of *C. difficile* spores

*C. difficile* isolates were first cultivated on BHIS agar plates supplemented with cycloserine (250 µg/ml) and cefoxitin (8 µg/ml). Overnight cultures of *C. difficile* were prepared and 100 µl of these cultures was spread onto BHIS agar. These plates were then incubated at 37°C under anaerobic conditions for 5 days, to allow for efficient sporulation [12], [16]. After 5 days, all spores and vegetative cells were harvested from the plates using cell scrapers (Costar®, Corning Incorporated, NY, USA) and suspended in dH_2_O before incubation overnight at 4°C to encourage the release of the spore from the mother cell [20]. Spore suspensions were then washed at least ten times in dH_2_O (16 000× *g*, 4 min). Following each centrifugation step the supernatant and top layer of the pellet, containing vegetative cells and cell debris, was carefully removed. After at least ten washes, spores were re-suspended in dH_2_O and checked for purity using phase-contrast microscopy. Spore suspensions, free of vegetative cells and cell debris, were stored at −20°C before use.

### Measurement of spore germination and outgrowth by colony formation

To measure the ability of *C. difficile* spores to complete germination and form colonies, spore suspensions were heat treated at 60°C for 25 min to kill any vegetative cells and then incubated in either i) BHIS; ii) BHIS supplemented with 0.1% (w/v) taurocholate; iii) BHIS supplemented with both 0.1% (w/v) taurocholate and 2 mM chenodeoxycholate; or iv) dH_2_O for 2 h. Following this incubation, the samples were washed and re-suspended in phosphate-buffered saline. Samples were then serially diluted and plated onto unsupplemented BHIS plates. CFU/ml were enumerated after 24 h incubation. A *spo0A* mutant, in which the master regulator of sporulation has been inactivated [16], was used as a negative control for colony formation after heat treatment.

### Measurement of the germination of *C. difficile* spores by loss of optical density

Prior to any spectrophotometric experiments, spore suspensions were checked, using phase-contrast microscopy, for impurity and clumping which could interfere with the subsequent OD_600_ measurements. Spore suspensions which showed extensive impurity and/or clumping were excluded from analyses. Purified spores were heat treated at 60°C for 25 min to kill any vegetative cells. Spore suspensions were then adjusted to an OD_600_ of 1 and 450 µl of each suspension was used per measurement. Spores were centrifuged and re-suspended in either BHIS or dH_2_O, and then split into three wells of a 96-well plate (for triplicate measurements). To each well, an equivalent volume of the respective germinant solution was added and germination was measured over 2 h. Heat-treated spore suspensions were germinated in either i) BHIS; ii) BHIS supplemented with either 0.1% or 1% (w/v) taurocholate (dissolved in dH_2_O); or iii) BHIS supplemented with both 0.1% (w/v) taurocholate and 2 mM chenodeoxycholate (dissolved in Tris-buffer by pH adjustment with NaOH to pH 8.0). Germination was expressed as the loss of spore OD_600_ over time using an iMark™ Microplate Absorbance Reader, with 5 sec of shaking prior to each measurement to avoid adherence of spores to the wells of the microtitre plate. After measuring OD_600_ over 2 h at room temperature, spore suspensions were again examined using phase-contrast microscopy to identify clumping of spores during the incubation period. Spore suspensions that showed evidence of clumping were excluded from the analyses, as spore clumping can cause a rapid drop in optical density that may be misinterpreted as the initiation of germination.

### Measurement of the return of *C. difficile* spores to vegetative cell growth

The return of dormant *C. difficile* spores to vegetative cell growth was evaluated by measuring the change in OD_600_ over a 20 h period following a 2 h incubation of spores with germinants. First, spore suspensions were heat treated at 60°C for 25 min to kill any vegetative cells and then incubated at room temperature for 2 h in either BHIS alone, or BHIS supplemented with 0.1% (w/v) taurocholate. Following this incubation, the suspensions were centrifuged at 8000× *g* for 4 min and re-suspended in BHIS. The samples were then incubated at 37°C under anaerobic conditions and cell growth was measured by the change in OD_600_ over 20 h using a microplate reader (TECAN GENiosPro 96/384).

### Biological replicates and statistical analyses

All data presented in this manuscript represent the results of three independent experiments. Statistical analyses were carried out in GraphPad Prism using one-way analysis of variance with Tukey's *post hoc* compensation for multiple comparisons of individual isolates.
